# Long-Term Care Research in the Context of COVID-19 Pandemic: A Bibliometric Analysis

**DOI:** 10.3390/healthcare11091248

**Published:** 2023-04-27

**Authors:** Zhaohui Sun, Lulu Chai, Ran Ma

**Affiliations:** Department of Law and Political Science, North China Electric Power University, Baoding 071003, China; 51952388@ncepu.edu.cn (Z.S.); 220192219023@ncepu.edu.cn (L.C.)

**Keywords:** long-term care, COVID-19 pandemic, CiteSpace, visualization

## Abstract

Despite the increasing awareness of long-term care (LTC) research after the outbreak of COVID-19 pandemic, little attention was given to quantitatively describe the evolution of the research field during this period. A total of 1024 articles retrieved from the Web of Science Core Collection database were systematically analyzed using CiteSpace visualization software. The overall characteristics analysis showed that, in the context of the pandemic, attention to LTC research increased significantly—over 800 articles were published in the past two years. The USA, Canada, Italy, and England formed the leading LTC research group, which was consistent with the conclusions of existing bibliometric studies on LTC research before the outbreak. A rigorous analysis based on a dual perspective of references and keywords was applied to reveal that, compared with previous studies, in the context of the pandemic, the focus shifted from the mental and physical health status of older adults in need of LTC to the impact of the pandemic on those of older adults in LTC facilities, from the prevention of general epidemics to the prevention and response of significant public health emergencies, from providing and paying for LTC to strategies for LTC facilities to improve the quality of LTC and well-being of their residents during the pandemic. These findings can provide help and reference for academics, civil folks, and LTC practitioners, as well as help with the sustainable development of LTC research in the context of COVID-19 pandemic.

## 1. Introduction

The COVID-19 pandemic disrupted the lives and health of people in an unprecedented way. The elderly population, especially those living in nursing homes and long-term care (LTC) facilities, were hit hardest by this pandemic. According to the data of the World Health Organization (WHO), as of 21 February 2023, more than 6.85 million patients died around the world, and the average age of the patients reported by each country was over 70 years old [1,2]. The nursing home COVID-19 data dashboard released by the Centers for Disease Control and Prevention (CDC) showed that the average weekly number of confirmed cases in nursing homes was 9967 from June 2020 to December 2022 [3]. Additionally, data shows that, in the USA, about 8% of people who lived in LTC facilities died of COVID-19—nearly 1 in 12, and for nursing homes alone, the figure was nearly 1 in 10 [4]. These facts attracted extensive attention from researchers around the world to the LTC research in the pandemic, and a wealth of research results were obtained.

Previous LTC studies covered the research areas of geriatrics and gerontology, nursing, public health, health care sciences and services, economics, and so on [5]. However, after the outbreak of COVID-19, scholars paid more attention to infectious diseases, immunology, and medicine research experiments, in addition to the above topics. Clearly, the pandemic, to a certain extent, contributed to make the research of LTC more complicated and multidisciplinary. Thus, it is necessary to discuss the influence of COVID-19 on the LTC research, and to summarize the status quo, evolution trends, and new frontiers of this research in the context of the epidemic. Existing LTC reviews after the outbreak of the pandemic focused mainly on specific subfields and themes. For example, Thompson et al. presented the latest data regarding the COVID-19 spread in LTC facilities worldwide, identifying causes and possible solutions that would limit the outbreaks in the elderly [6]; Konetzka et al. reviewed the empirical evidence on LTC facility characteristics associated with COVID-19 cases and deaths [7]; Hu et al. reviewed the current status of COVID-19 on the patients with several neurodegenerative diseases and summarized the potential mechanisms of SARS-CoV-2 infection in the pathogenesis of those diseases [8]. This narrow focus helped deepen our understanding of specific facets of LTC in the pandemic, but the resulting fragmentation of LTC research prevents us from seeing the overall picture.

This research aims to conduct a comprehensive and in-depth scientific evaluation of LTC research in the context of COVID-19 pandemic through quantitative research. Specifically, our review is guided by two key goals: to depict the characteristics of LTC research publication outputs in the pandemic from the perspectives of publication and citation and to identify how research hotspots and trends changed compared with previous studies. Our study differs from previous research in three ways: we performed a topic search of all publications using “COVID” and “long-term care” as keywords instead of focusing on a particular aspect; we used a broad literature search via the Web of Science Core Collection (WoSCC) database for all relevant articles instead of focusing on the key articles from specified journals; and we made a comparation of hotspots and trends in this field with the research before the pandemic.

## 2. Materials and Methods

### 2.1. CiteSpace

CiteSpace version 5.8. R3 was selected as the tool for visualizing bibliographic records. This software was first developed by Dr. Chaomei Chen at Drexel University (Philadelphia, PA, USA). CiteSpace makes a statistical analysis of scientific references in different scientific fields based on the technologies of computer graphics, images, intelligent technology to convert these references into visualization graphs. Specifically, it was developed to map knowledge domains, clarify the relationships between different disciplines, assess the status of research, reveal hot research topics, and forecast emerging trends to help the readers directly visualize research evolution and development processes. According to Chen, visualization knowledge maps consist of nodes and links. Different nodes represent elements such as authors, institutions, countries, keywords, and cited references, and links between nodes represent relationships of collaboration, co-occurrence, or co-citations. In addition, the color of the links indicates the time of the first co-occurrence or co-citation between nodes, and the thickness of a link shows the strength of connection between two nodes [9].

In this study, our analysis primarily depended on three types of bibliophilic techniques applied using CiteSpace. Firstly, collaboration network analysis is critical to understanding scholarly communication and knowledge diffusion. It evaluates the published contributions and academic impact of countries, institutions, and authors through a visual network of scientific collaboration [10]. Secondly, document co-citation analysis is a statistical method that is used to studies of the structure, dynamics, and paradigm developments of a given research field. In particular, co-citation analysis is mostly conducted by co-citation clusters, which helps to identify frequently co-cited papers more credibly and provides important insights into knowledge domains. Thirdly, keyword co-occurrence analysis processes keywords or terms that are extracted from the title, abstract, or body of literature to establish word co-occurrence relationships. It is an effective way to show emerging trends and track topics of research over time, which can help researchers identify evolving research frontiers relating to a knowledge domain. Significantly, CiteSpace can both construct bibliometric networks for different phases and visualize burst terms and high betweenness centrality to identify emerging trends, radical changes, and turning points in research [10].

### 2.2. Bibliographic Records

The input data for this study were taken from Science Citation Index Expanded (SCI-E) and Social Sciences Citation Index (SSCI) via the WoSCC database. It covers over 10,000 leading journals worldwide and provides powerful access to bibliographic information and citation information pertaining to research articles published, which is considered to be the ideal data source for actinometric research.

The data were collected on 31 December 2022 by two researchers simultaneously using the keywords retrieval conditions shown in Table 1 and the literature type ‘Articles’ and ‘Review Articles’ were selected. There are three steps in collecting data for CiteSpace analysis. First of all, we performed a topic search of all publications that contained these words in title, abstract, and keywords and obtained 1028 literature. In addition, after reading the title and abstract of the obtained literature, we removed four repeat bibliography and then, the original texts of all the documents were downloaded, verified one by one, and cross-checked. Finally, according to our retrieval results, the research team members reached the same conclusion on the screening results, and the further validity test showed that the top 200 most-cited articles were closely related to LTC and COVID, indicating that our retrieval strategies were appropriate. Finally, a total of 1024 literature, published from 01 January 2020 to 31 December 2022, were retrieved, including title, author, abstract, keywords, references, and other information.

## 3. Results and Discussion

### 3.1. Characteristics of Global Publication Outputs

**Figure 1:**
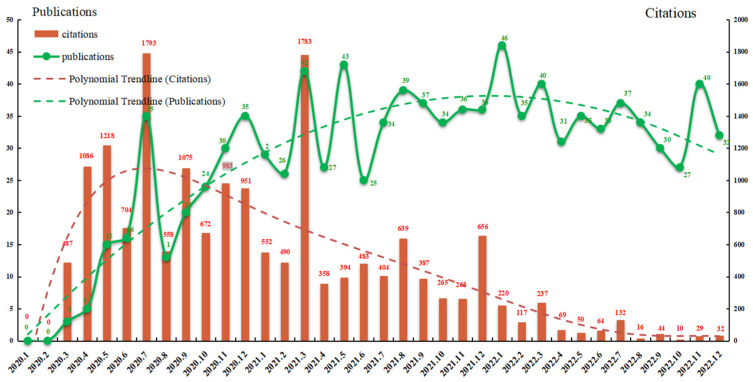
Month-wise distribution of publications and citations from 2020 to 2022.

Based on the collected data, there were 939 articles and 85 review articles published in seven different languages. English (1002) was the predominant language accounting for 97.85%, while less than 3% were published in other languages. It is undeniable that English literature is expected to take up a higher and higher proportion in the future literature output, because more and more of the journals included in WoSCC choose to publish in English. These articles cover several research areas, including health care sciences services, infectious diseases, public environmental occupational health, geriatrics gerontology, respiratory system, general internal medicine, nursing, immunology, psychology, and sociology. The distribution of research areas suggested LTC research became more interdisciplinary after the outbreak of COVID-19 epidemic. Moreover, it was particularly noticeable that epidemiology and prevention control played an important role in 2020–2022. To better understand the research status of LTC in the context of COVID-19 pandemic, the trend of the publications and citations is shown in Figure 1. The green points represent the number of citations per month and exhibit a rapid rise, and then, a fluctuating decline. The bar graphs illustrate the monthly publication counts, showing a trend of wave-growing. Additionally, two trendlines were identified by fitting a polynomial to the data, as revealed by the dotted lines.

- Preparation phase (January 2020–June 2020). At the beginning of this stage, no relevant article was published in January and February. Despite the huge impact of COVID-19 on LTC, little literature had been published at this stage, probably because the relative impact of COVID-19 on LTC research was still in the exploratory stage. Remarkably, although only a few articles were published in March, April, and May in 2020, these articles had a high number of citations, which proved their influence in the LTC research field. It also revealed that there was a strong accumulation of citations, and the earlier the papers published, the higher the citations.
- Fluctuating growth phase (July 2020–June 2021). The number of publications maintained a trend of continuous fluctuating growth during this period, and reached the peak in December 2020, March 2021, and May 2021, respectively. The number of articles increased more rapidly than before, and more than 345 articles were published. It is worth noting that the biggest citation burst was found in July 2020 (1793) and March 2021 (1783), and the number of citations exceeded 500 in most months in this period, indicating that scholars had paid great attention to LTC research and produced many high-quality papers.
- Stable development phase (July 2021–December 2022). Since July 2021, LTC research in the context of the pandemic had become one of the most significant concerns among policy makers, related scientists, international organizations, and national organizations. The number of publications remained at a high level, with an average of more than 30, but fluctuated slightly. In consideration of the citation trendline, the number of citations of the papers continued to decline. Such a decrease can be explained by taking into account that citations of newly published articles are subject to the time lag and have less chance of being cited.

### 3.2. Collaboration Network Analysis

In general, the number of outputs is associated with the national research scale, the number of research institutions, quantity, and quality of authors’ publication, and the proportion of those that have a LTC research focus in the context of COVID-19 pandemic. In this section, we depicted the knowledge maps to identify major contributors in LTC research in the pandemic on the analyses of collaboration networks of countries/regions, institutions and authors. The analyses were conducted as follows.

#### 3.2.1. Network of Countries/Regions

**Figure 2:**
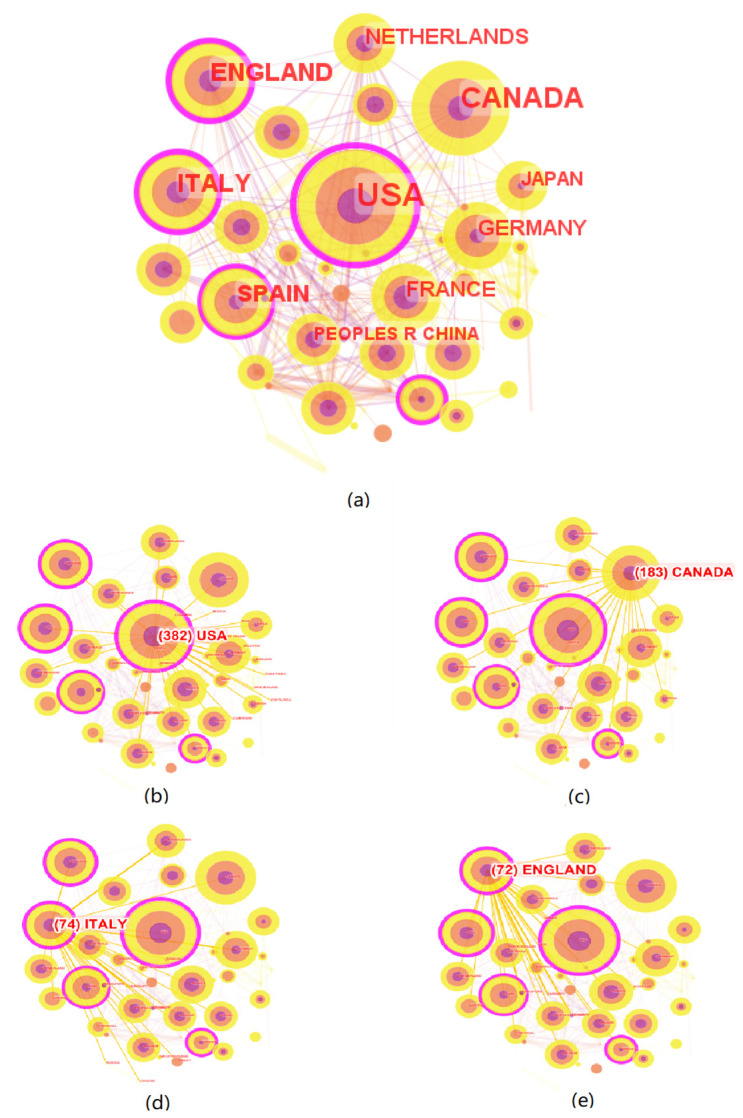
The visualization map of countries/regions. (**a**) All countries/regions, (**b**) USA, (**c**) Canada, (**d**) Italy, (**e**) England.

Analysis of collaboration among countries used the following parameters in CiteSpace: (1) time slice from 2020 to 2022; (2) years per slice = 1; (3) term source = title/abstract/author keywords/keywords plus; (4) node type = country; (5) pruning = none; (6) select the criteria g-index, k = 25. As shown in Figure 2, the research network, containing 59 nodes and 284 links, was obtained by CiteSpace. Each node was a country or a region and each link represented the collaborative relationship between two nodes. Generally, the node size represents the publication volume, and the thickness of connecting lines between countries demonstrated the intensity of cooperation. It was worth noting that nodes with high betweenness centrality (>0.1) in the network were indicated by purple rings which connected more links.

In order to obtain more information about countries/regions, the ranking of the top 10 contributors by counts of publications was established and is shown in Table 2. In general, the total number of papers published by these countries accounts for 98.70% of all papers. Specifically, two North American countries, the United States and Canada, which have the language advantage of publishing papers in international journals, ranked first and second with 382 and 183 publications, respectively. Six European countries, Italy, England, Spain, Germany, the Netherlands, and France, ranked 3rd–8th. Two East Asian countries, Japan and China, ranked ninth and tenth. The USA, Canada, Italy, and England appeared to be the leading countries in terms of publication volume and centrality, almost identical to the results before the pandemic, indicating the continued concern for LTC research in these countries after the outbreak of COVID-19 pandemic. Similarly, in line with pre-pandemic results, China was the only developing country in the most productive countries.

As can be seen in Figure 2, the USA played core roles in the cooperation network with purple rings. We further mapped the network of cooperation between the top four countries; according to Figure 2b–e, the cooperation network centered on the USA, Italy, and England had large scopes, suggesting that they had high international status and strong international cooperation in the field of LTC research during the COVID-19 pandemic. However, limited cooperation was found in Canada, which was reflected in the low betweenness centrality and the loose cooperation network in this country. Moreover, prolific countries ranked 4–10 all had a centrality of less than 0.1, indicating that they had less cooperation with other countries. However, in terms of coping with the challenges of globalization and cooperating to fight the epidemic, the need for international exchanges and cooperation in this field is more urgent than ever. Therefore, efforts should be made to develop international cooperation in exploring the LTC research areas in the context of the pandemic, and to build a closer national cooperation network.

#### 3.2.2. Network of Institutions

**Figure 3:**
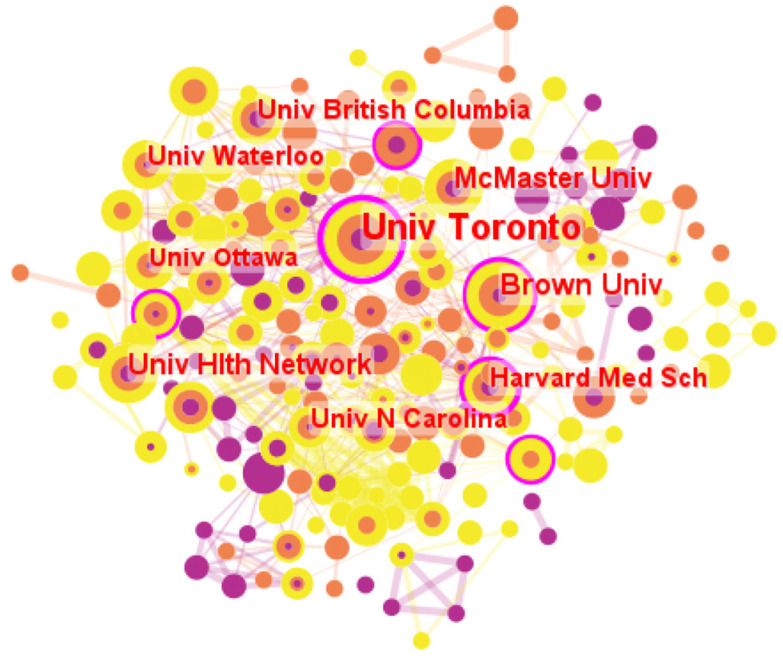
The visualization map of institutions.

Characteristics of the institutional cooperation network map reflected the research capacity of institutions to a certain extent. The contribution and influence of each research institution could be effectively distinguished through statistical analysis. In this section, the parameters in CiteSpace were kept the same, except for node type being changed from “Country” to “Institution”. The visualization map comprising 208 institutions and 698 collaboration links between institutions was shown in Figure 3.

As shown in Figure 3, it seems that the top ten most productive institutions were mainly from Canada and the USA, including seven Canadian institutions (six universities and one university organization) and three American universities. The results further confirmed the importance of institutions in North America for LTC research during the pandemic. In terms of publication counts, the top five institutions issued 203 articles, accounting for 19.82% of the total. To be specific, the University of Toronto ranked first with 80 papers, followed by Brown University (39), McMaster University (29), University Health Network (29), and Harvard Medical School (26).

The density of institutional cooperation network was 0.0324, which revealed that the cooperation between research institutions was not strong, an extensive and close cooperation network had not been formed. Therefore, the cooperation between institutions needed to be strengthened. According to Figure 3, the node with a purple circle indicated that it had a betweenness centrality exceeding 0.1. Moreover, the thicker the circle, the stronger the centrality, that is, the higher the connection ability in the network. Table 3 lists the top four productive institutions with the centrality over 0.1. As seen in Table 3, remarkable betweenness centrality values appeared in the University of Toronto (0.41, 80), Harvard Medical School (0.24, 26), Johns Hopkins University (0.16, 16), and Brown University (0.26, 27). Additionally, authors from these institutions formed a close communication and cooperation network and had greater scientific research output and contribution. In particular, the institution with the largest contribution was the University of Toronto, a public federal research university and one of the top institutions in the world, which conducted a series of studies on the infection, detection, transmission, and control of SARS-CoV-2 in LTC homes in Canada. It is noteworthy that although the publication counts of Emory University (0.14, 10) and Oxford University (0.15, 10) were less than 15, the betweenness centrality values of them were all above 0.1, indicating that these institutions had strong international communication strength and the LTC research potential under the epidemic.

#### 3.2.3. Network of Authors

**Figure 4:**
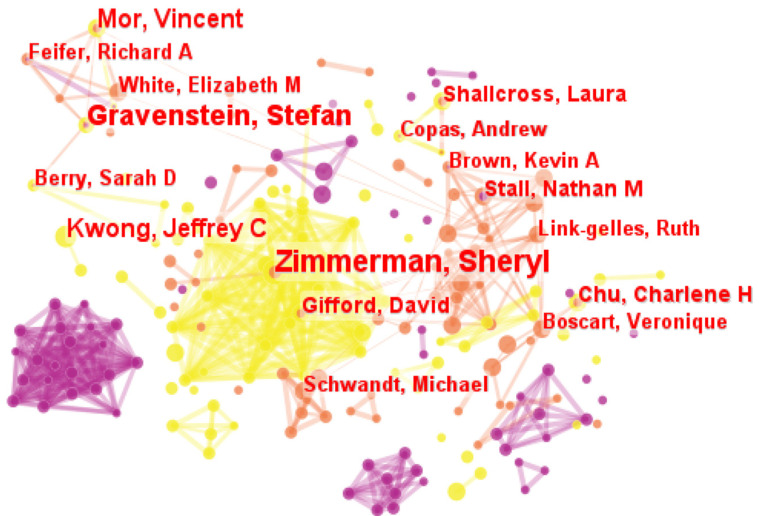
The visualization map of authors.

We found potential collaboration relationships through co-citation analysis in the author collaborative network. The parameters in CiteSpace were kept the same except node type being changed from “Institution” to “Author”. Figure 4 depicted the distribution of the publications by authors and the collaboration between them, consisting of 197 nodes and 563 collaborative links. According to Figure 4, the larger a node was, the more articles the author published, namely, the greater contribution the author made.

It seems that the authors tended to collaborate with a single, highly productive author, thus forming several co-author clusters. Table 4 listed the top six productive authors and the co-author clusters they formed. As seen in Table 4, there were three main co-author clusters, with Zimmerman, Gravenstein, and Stall as the central authors, respectively. The authors in the Zimmerman cluster mainly conducted research on environmental detection, outbreak management, and infection control of COVID-19 pandemic in LTC facilities, the authors in the cluster of Gravenstein paid close attention to the vaccination and SARS-CoV-2 infection in nursing homes, and authors in the Stall cluster concentrated more on the risk of COVID-19 outbreak in nursing homes and the visitor policy during the pandemic. However, Kwong had no collaboration with other authors and only focused on independent research. In general, according to the distribution of authors in the map, it can be found that LTC research in the context of the epidemic showed the characteristics of “partial concentration and overall dispersion”. Additionally, all authors had low betweenness centrality (<0.1), revealing that the influence and cohesion of existing scholars needed to be improved.

According to the previous study [5], Mor and Zimmerman were the most influential researchers on LTC research before the epidemic. As can be seen in Table 4, they still had great contributions to this field. Especially, Zimmerman became the most productive author in the field after the pandemic with the publication counts of thirteen. She is a distinguished professor of social work and public health at Kenan Flagler, and the interdisciplinary center in aging research in the University of North Carolina System. Her main concerns were as follows: racial disparities in health outcomes during the pandemic [11], the care model of Green House/small nursing homes post-COVID [12], impact of the epidemic on LTC models [13], clinical research in nursing homes to address prevention and treatment of COVID-19 [14]. The second most influential author was Gravenstein, a geriatrician, the David S. Greer professor of geriatrics, as well as professor in the departments of medicine and health services policy and practice at Brown’s schools of medicine and public health. Dr. Gravenstein had a long-standing interest in immunity, inflammation, cardiovascular outcomes, and aging, especially in the context of vaccines and the long-term care setting, which was the topic of the majority of his publications. Beginning in 2020, he became active in several projects related to COVID in long-term care, including vaccines, viral infections, etc [15,16,17,18].

### 3.3. Document Co-Citation and Keyword Co-Occurrence Analysis of LTC Field in the Pandemic

In this section, we depicted the knowledge maps to identify hot topics and research frontiers in LTC research in the context of COVID-19 pandemic on the analyses of cited references and co-occurring keywords. The analyses were conducted as follows.

#### 3.3.1. Document Co-Citation Network

We conducted document co-citation analysis to define the underlying intellectual structures of LTC domain in the context of COVID-19 pandemic. In this process, co-citation clusters were also identified, which could reflect the evolution process of scientific activity in this field. The following parameters in CiteSpace were used: (1) time slice from 2020 to 2022; (2) years per slice = 1; (3) term source = title/abstract/author keywords/keywords plus; (4) node type = reference; (5) pruning = none; (6) select the filtering condition G-index, k= 25. After running CiteSpace, a co-citation cluster network which contained 391 nodes and 1594 links was visualized. Each node represented a document in the field of LTC research and was labeled with the author’s name and the publication year, whereas each link between nodes reflected the co-citation relationship between the two corresponding documents. To produce this graph, a total of nine co-citation clusters were identified using the LLR algorithm, each of which was a group of tightly coupled references representing a thematic concentration in the bibliographic landscapes.

**Figure 5:**
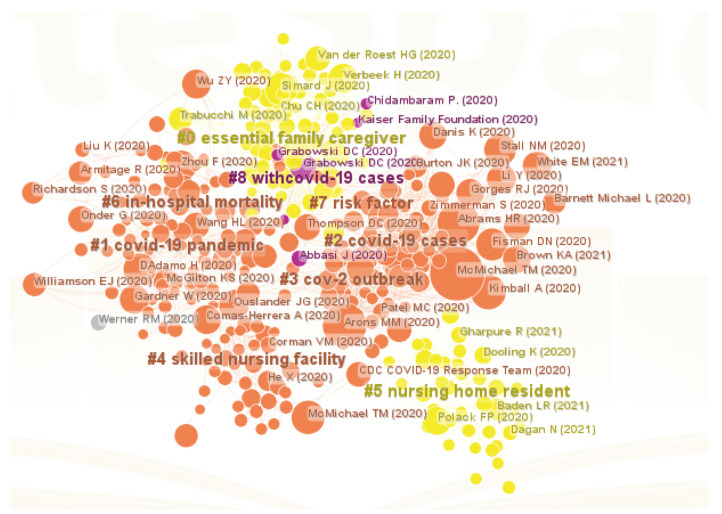
The visualization map of references. Note: # represents a knowledge cluster [6,12,15,19,20,21,22,23,24,25,26,27,28,29,30,31,32,33,34,35,36,37,38,39,40,41,42,43,44,45,46,47,48,49,50,51,52,53,54,55,56,57,58,59,60,61,62,63,64].

Generally, a highly cited article means a landmark of the domain. We summarized the top 18 most-cited references listed in Table 4, from No. 1 to No. 18, assigned to seven clusters. Moreover, two references with high betweenness centrality, as indicated by purple rings in Figure 5, were presented as Nos. 3–4. Ten references (i.e., No. 1, No. 7, No. 11, and Nos. 19–26) with the strongest bursts in the group of references that started to burst at the same time can be adopted to disclose the LTC research trends in the context of COVID-19 pandemic. Furthermore, we investigated the top five references in each cluster by cited counts. Table 5 lists detailed descriptions of 49 representative references.

The first largest cluster (#0) contained 70 references labeled as “essential family caregiver”. Essential family caregivers are typically family members or friends who were a steady presence at a loved one’s facility, providing companionship and help with daily activities such as eating, bathing, and grooming. The outbreak of COVID-19 in LTC facilities resulted in severe impact on nursing home residents and staff, posing great challenges to the care of older people [25,31,45,46]. Additionally, the shortage of medical resources and staff [25] made essential family caregivers critical to improving the safety, health, and well-being of older people in nursing homes in the wake of COVID-19 [45]. A better balance between physical safety and well-being could be achieved through more sensible visitor policies during the pandemic, as social isolation is a serious health threat for older residents and increases the risk of mortality [44].

Cluster #1 contained 65 references and was labeled as “COVID-19 pandemic”. COVID-19 pandemic rapidly affected mortality worldwide [50], and most of those who died were older adults, especially those with underlying health problems [47,50]. In order to shield the vulnerable elderly, worldwide countries enforced lockdowns, curfews, and social isolation to mitigate the spread of the pandemic [24,47,49]. Social isolation among older adults might reduce transmission, but it can have an impact on the mental health of the elderly and become a serious public health problem [49]. At the same time, this pandemic highlighted the long-standing structural deficiencies affecting the nursing homes, which was an opportunity to provide some considerations for nursing home leaders and regulators to support the health and well-being of nursing home staff and residents [48].

The third largest cluster (#2) had 55 members and was labeled as “COVID-19 cases”. Most references in this cluster focused on the statistical information of COVID-19 cases or deaths through publicly available data and found that larger facility size, urban location, greater percentage of African American residents, non-chain status, and state [21], higher registered nurse staffing and quality ratings [15], reducing overcrowding in nursing homes [51], higher nurse aide hours, and total nursing hours [52] might help contain the number of cases and deaths. Additionally, studies showed that nursing home staff were working under complex and stressful circumstances during the COVID-19 pandemic. These challenges added significant burden to an already strained and vulnerable workforce and are likely to contribute to increased burnout, turnover, and staff shortages in the long term, leading to increased COVID-19 cases and deaths [32].

There are other clusters worth mentioning. References in cluster #3 had a common concern for the transmission, testing, infection control of SARS-CoV-2 [19,20,22,29,42]. The most active citer to cluster #4 [23,30,41,53,54] was McMicheal [23], who suggested that implementation of public health measures targeting vulnerable populations such as residents of LTC facilities and health care personnel, providing information for patients and families as well as communicating more broadly to all stakeholders will be critical to manage the pandemic. Five references in cluster #5 were mainly about the safety and efficacy of SARS-CoV-2 Vaccine [38,39,43,55,56]. References in cluster #6 reflected a common theme—the mortality and characteristics of patients dying in relation to COVID-19 [28,34,36,37,57]. The common topic of cluster #7 and cluster 8 was the risks of COVID-19 to nursing homes [6,58,59,60,61,62,63,64]. Meanwhile, post-acute care for COVID-19 attracted much attention [12].

Obviously, the COVID-19 epidemic had an impact on the cognition of the importance of LTC for the elderly and attracted more attention. In comparison with previous studies, we found that dementia care in nursing homes, quality of care, disease prevention and control, healthcare providers, and LTC facilities and residents were still the emphases of LTC research during the pandemic. However, studies before COVID-19 pandemic mainly concentrated on health status, mortality, database application, providing and paying for LTC, and frailty in elderly people [5]. What made LTC research in the pandemic different was that the research paid more attention to the impact of the pandemic on LTC facilities and residents, including the effects on physical health, mental health, and human rights of older adults, how LTC facilities cope with COVID-19 epidemic and infection prevention and control strategies. In general, the focus shifted from the mental and physical health status of older adults in need of LTC to the impact of the pandemic on those of older adults in LTC facilities, from the prevention of general epidemics to the prevention and response of significant public health emergencies, from providing and paying for LTC to improving management and developing strategies for LTC facilities to improve the quality of LTC and well-being of their residents in the context of the pandemic.

**Figure 6:**
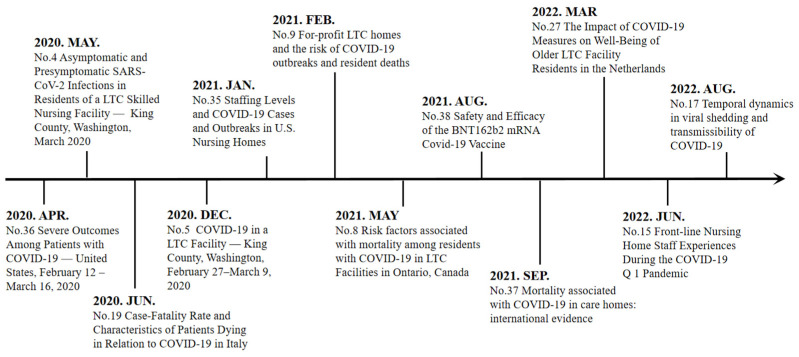
Timeline of the 11 burst references.

In order to obtain an impression of evolution of research fronts in this research, we further focused on 11 burst references (i.e., Nos. 4–5, Nos. 8–9, No. 15, No. 17, No. 19, No. 27, and Nos. 35–38) in Table 5. An article with citation burst means it received an increased attention in a certain period of time. Figure 6 shows a timeline view of 11 burst references with their respective research foci. The LTC research trends at different times were revealed as three stages: in the early stage from January 2020 to December 2020, research focused on COVID-19 infection prevention and control strategies in LTC facilities; in the second stage from January 2021 to May 2021, focus shifted to COVID-19 cases and deaths in LTC facilities; in the third stage from June 2021 to December 2022, research of the COVID-19 virus, vaccine, and nursing homes received increased attention. In short, with the outbreak of COVID-19, research on LTC became more in-depth and diversified, mainly reflected in the significant increase in the proportion of database-based empirical research and survey research.

#### 3.3.2. Keyword Co-Occurrence Network

**Figure 7:**
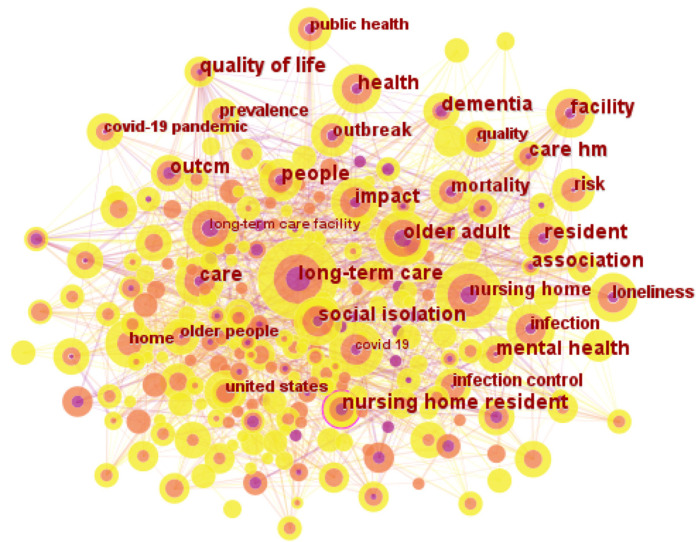
Network of co-occurring keywords.

An analysis of keywords can help us identify hot topics of LTC research in the context of COVID-19 pandemic. Figure 7 shows the co-occurring keywords network produced by CiteSpace. The parameters in CiteSpace remained the same except the node type being changed from “reference” to “keyword”. The network contains 275 nodes and 1270 links in total from 2020 to 2022. In the figure, node size represents the frequency of the keyword in the record, and lines that connect nodes are co-occurred links. Furthermore, we merged the similar keywords that are in fact variants of the same entity, for example, “nursing hm” was merged into “nursing home”, “long term care” was merged into “long-term care”.

In particular, high-frequency keywords can reflect the research heat and keywords with high centrality represent major intellectual turning points and connecting more other keywords. Therefore, we identified 26 representative keywords in terms of the counts and betweenness centralities in Table 6. The top 25 keywords from No. 1 to No. 25 had a co-occurrence frequency over 25. Additionally, one keyword No. 26 had high centrality.

The top two on the list in terms of co-occurrence frequency were “long-term care” (299) and “nursing home” (230). The outbreak of COVID-19 in nursing homes resulted in a high mortality rate and had a significant impact on the daily management of nursing homes, making it a hot topic for LTC research in the pandemic. Keywords with high centrality were observed in “nursing home residents”, which represented major intellectual turning points linking different keywords with significantly influenced LTC research development.

As for these 26 high-frequency keywords, according to previous scientometric studies, they can be directly regarded as LTC research hotspots. To focus on the major issues, we identified the hot research topics by integrating 26 high-frequency keywords and considering the co-occurring keywords shown in Figure 7. The resulting four main hot spots were as follows:

- Nursing homes and residents was extracted using keywords “nursing home”, “long-term care”, “long-term care facility”, “resident”, “impact”, “facility”, “United States”, and “nursing home residents”. At the first outbreak of COVID-19, nursing homes became the hotbed for it [6]. According to the data, more than one-third of COVID-19 death happened at nursing homes in the United States, even in some states, the proportion is more than one-half [54]. Viral infection and COVID-19 disease are prevalent among nursing home residents [29], due to their congregant living environments, greater likelihood of being exposed to asymptomatic and pre-symptomatic care providers, and difficulty in effectively implementing infection prevention and control practices [22]. This pandemic had put both nursing homes and residents at acute risk highlighting the limited resources many facilities had in dealing with crises of this magnitude [29].
- Older people in need of LTC in the pandemic was identified using nine keywords “long-term care”, “older adult”, “COVID-19”, “care”, “health”, “infection”, “risk”, “older people”, “mortality”, “dementia”, and “mental health”. Older people were the group of most susceptible to COVID-19, adding further difficulties to their LTC [25,45,46]. This was mainly due to the higher incidence of immune dysfunction, chronic diseases, and disabilities in the elderly, which could develop a more severe form of the disease, and further lead to increasing mortality [22,25]. Furthermore, as for the individuals with Alzheimer’s disease and related dementia, the pandemic disrupted not only the basic routines, but also the LTC that promote their physical and mental health [12]. However, it is particularly distressing that few care to frail and needy older people could be offered [46]. Awareness of clinical differences of COVID-19 in this population, quickly initiating appropriate behaviors to care for the infected, and preventive interventions would help better LTC for the elderly in this crisis [25].
- Infection prevention and control strategies was extracted using the keywords “COVID-19”, “infection”, “outbreak”, “prevalence”, and “public health”. There was a consensus that all patients involved in LTC should take proactive steps to prevent the epidemic [20,22]. To begin with, for residents of LTC facilities, frequent hand washing, universal use of face masks, and reducing contact were effective ways to control the spread of the epidemic [22,58,59]. For all facilities providing LTC, strategies include restricting nonessential personnel from entering the facility [20,22]; additional prevention measures for asymptomatic or pre-symptomatic [30,54]; increasing in payments to direct caregivers [29]; and continuous communication with residents and family members [12,45]. Government departments and national health departments might need to enhance the infection control capacity [12], invest in public health infrastructure [29], improve international surveillance, cooperation, coordination, and communication, as well as be better prepared to respond to future new public health threats [33].
- Social isolation and loneliness comprised eight representative keywords “COVID-19”, “infection”, “risk”, “quality”, “social isolation”, “loneliness”, “mental health”, and “home”. Social isolation and loneliness caused by quarantine policies adopted to prevent the spread of the epidemic take a serious toll on the physical and mental health of older people in need of LTC [34,49]. Personal interactions were meaningful activities and are crucial to improve the quality of LTC [30]. Many older people in need of LTC were socially isolated and lonely, depending on frequent visits from family and friends to socialize with them [34]. However, quarantine policies during the pandemic prevented these visits, making older people feel increasingly lonely, abandoned, and despondent [34]. At the same time, it could also cause anxiety and emotional trauma to families and others who could not visit their loved ones [29]. Therefore, it is important to recognize the role that family members play as partners in LTC of the elderly and develop visitor policies in LTC facilities during the pandemic [30].

On the whole, the change of research hotspots identified by keywords after the outbreak of the pandemic is similar to the trend shown by cited references. According to previous study [5], the hot topics of LTC research mainly included dementia, quality of care, prevalence, and risk factors and mortality. While, after the outbreak of the pandemic, the hot topics of the research became the impact of the pandemic on LTC including the crisis of limited resources in nursing homes, the harm to the physical and mental health of the elderly, the increasing need for LTC among the elderly, the lack of effective infection prevention and control strategies, as well as the loneliness caused by policy of social isolation. In general, the pandemic made scholars more focused on the health services and right to survival and development of the elderly. Improving LTC in the context of the pandemic will help improve the health of older people and contribute to the prevention and treatment of COVID-19 in the elderly.

## 4. Conclusions

In this paper, we drew on bibliometric data relating to 1024 journal articles listed on the WoSCC database. Scientific output and citations of LTC research in the context of COVID-19 pandemic and the collaboration networks were visualized to examine the current status, development, and major contributors to the research. Document co-citation analysis and keyword co-occurrence analysis enabled us to explore hot topics and new frontiers in LTC research during the pandemic, and to find out the changes of research focus by comparing with the research before the epidemic. The specific findings were as follows:

Firstly, LTC research in the context of COVID-19 pandemic developed steadily with fruitful achievements. From the number of publications, it increased slowly in the first six months and broke out in July, then decreased, and then, kept the increasing trend with fluctuations. After July 2021, the number of publications remained at a high level, with an average of more than 30. In addition, the trendline of publication volume showed a trend of slow growth followed by a steady growth, and then, a slight decline. In consideration of the number of citations, there was a significant increase at first, and the outbreak occurred in July 2020 and March 2021, and then, decreased with fluctuation. Additionally, the trendline showed a trend of downward. Generally speaking, a significant increase was seen in the number of publications in 2021 and 2022, with over 800 papers published, indicating that researchers were evolving LTC research in the pandemic and this trend would continue.

Secondly, the major contributors provided a wealth of results for LTC research during the pandemic of great significance. Nevertheless, international collaboration among them should be strengthened. The USA, Canada, Italy, and England appeared to be the leading countries in terms of publication volume and centrality, which was consistent with the conclusions of existing bibliometric studies on LTC research before the outbreak [5]. In addition, the top 10 most productive institutions mostly came from Canada and the USA, which proved to be the main research forces in this field. The University of Toronto was identified as the most influential institution in this field based on publication counts and betweenness centrality, and Zimmerman and Gravenstein were the most productive and high-impact authors. However, collaboration networks of major contributors showed that the cooperation between them was not strong, and efforts should be made to form extensive and close cooperation networks when exploring LTC research areas in the context of the pandemic.

Thirdly, consistent results were found from the current analysis of the knowledge maps of references and keywords. It was revealed that the common LTC research hot topics in the COVID-19 pandemic were impacts of the pandemic on LTC facilities and residents including the effects on physical health, mental health, and human rights of older adults, loneliness caused by policy of social isolation, how LTC facilities cope with COVID-19 and infection prevention and control strategies. Additionally, the trends of LTC evolution showed three stages: the first stage from January 2020 to December 2020, where research was primarily focused on COVID-19 infection prevention and control strategies in LTC facilities; the second stage from January 2021 to May 2021, where the focus shifted to aspects of epidemiological of COVID-19 in different countries; and the third stage from June 2021 to December 2022, where research of the COVID-19 virus, vaccine, and nursing homes received increased attention. Compared with previous studies, with the outbreak of COVID-19, the focus shifted from the mental and physical health status of older adults in need of LTC to the impact of the pandemic on those of older adults in LTC facilities, from the prevention of general epidemics to the prevention and response of significant public health emergencies, from providing and paying for LTC to improving management and developing strategies for LTC facilities to improve the quality of LTC and well-being of their residents in the context of the pandemic.

In conclusion, this paper provides valuable information to LTC researchers to identify new perspectives in the context of COVID-19 pandemic concerning major countries/regions, institutions, researchers, hot topics, evolution trends, and new research frontiers, which should be strongly focused by academics, civil folks, and LTC practitioners. Moreover, it provided a new scientific visualization method to construct concept networks on LTC and contribute to the integration of LTC researches in the pandemic. For LTC practitioners, this study presented accurate information regarding the key authors and institutions best suited to assist in developing LTC systems to better manage the COVID-19 pandemic and any other future epidemics. It should be noted that there are some limitations in our paper, such as we only collected bibliographic records on LTC from one database—the WoS. Future studies may carry out a broader study based on other databases to complement the preliminary results with the current study. In addition, it is necessary for us to analyze important domestic and international policies regarding LTC from every country involved in this pandemic to explore effective policies to deal with the epidemic.

## Figures and Tables

**Table 1:** Keyword search using terms related to LTC and COVID.

Main Keyword	Related Keywords
COVID	“2019-nCoV” OR “SARS-CoV-2” OR “Corona Virus *” OR “Coronavirus Disease 2019” OR “2019 Coronavirus Disease” OR “New coronavirus disease” OR “Novel coronavirus disease” OR “Novel corona virus *” OR “New corona virus *”
AND	
Long-term care	“Long term care”

* represents any group of characters, including no character.

**Table 2:** Most prolific countries/regions (2020–2022).

NO.	Count	Centrality	Country
1	382	0.56	USA
2	183	0.08	Canada
3	74	0.28	Italy
4	72	0.26	England
5	60	0.13	Spain
6	51	0.08	Germany
7	40	0.06	Netherlands
8	35	0.03	France
9	27	0.10	Japan
10	24	0.02	China

**Table 3:** Most prolific institutions with a centrality over 0.1 (2020–2022).

NO.	Count	Centrality	Institution	Country
1	80	0.41	University of Toronto	Canada
2	39	0.11	Brown University	USA
3	26	0.24	Harvard Medical School	USA
4	16	0.16	Johns Hopkins University	England

**Table 4:** Most prolific authors and co-author clusters (2020–2022).

NO.	Author	Publications	Author Clusters
1	Zimmerman, Sheryl	13	Zimmerman, Sheryl; Nace, David; Gifford, David; Schwandt Michael; Linkgelles, Ruth
2	Gravenstein, Stefan	11	Gravenstein, Stefan; Mor, Vincent; White, Elizabeth M; Blackman, Carolyn; Feifer, Richard A
3	Kwong, Jeffrey C	7	-
4	Mor, Vincent	7	Gravenstein, Stefan; Mor, Vincent; White, Elizabeth M; Blackman, Carolyn; Feifer, Richard A
5	Gifford, David	6	Zimmerman, Sheryl; Nace, David; Gifford, David; Schwandt Michael; Link-gelles, Ruth
6	Stall, Nathan M	6	Stall, Nathan M; Brown, Kevin A; Boscart, Veronique; Jones, Aaron; Costa, Andrew P Schwandt, Michael; Mckee, Geoff; Vijh, R; Harding J; Hayden, A; Lysyshyn, M

**Table 5:** Forty-nine representative references in terms of citations, centrality, and bursts.

No.	Count	Centrality	Strength	Reference	Year	Begin	End	Cluster ID
1	131	0.06	9.32	McMichael et al. [19]	2020	2020	2020	#3
2	84	0.05	2.22	Arons et al. [20]	2020	2020	2020	#3
3	83	0.11	0.00	Abrams [21]	2020	-	-	#2
4	55	0.11	1.89	Kimball [22]	2020	2020	2020	#3
5	49	0.02	2.28	McMichael [23]	2020	2020	2020	#4
6	46	0.03	0.00	Simard [24]	2020	-	-	#0
7	46	0.02	4.94	DAdamo [25]	2020	2020	2020	#1
8	44	0.02	0.00	Stall [26]	2020	-	-	#2
9	44	0.06	0.00	Fisman [27]	2020	-	-	#3
10	43	0.01	0.00	Zhou [28]	2020	-	-	#6
11	40	0.00	0.00	Ouslander [29]	2020	-	-	#3
12	39	0.03	0.00	Verbeek [30]	2020	-	-	#0
13	39	0.01	0.00	Comas [31]	2020	-	-	#4
14	38	0.01	0.00	White [15]	2021	-	-	#2
15	38	0.03	0.00	Li [32]	2020	-	-	#2
16	36	0.02	1.84	Wu [33]	2020	2020	2020	#6
17	36	0.03	3.25	Gardner [34]	2020	2020	2020	#7
18	35	0.03	0.00	Danis [35]	2020	-	-	#3
19	29	0.03	4.51	Onder [36]	2020	2020	2020	#6
20	8	0.01	4.11	Liu [37]	2020	2020	2020	#6
21	13	0.00	3.29	Dooling [38]	2020	2021	2022	#5
22	11	0.01	2.78	Ghanpure [39]	2021	2021	2022	#5
23	10	0.00	2.53	Werner [40]	2020	2021	2022	#4
24	10	0.01	2.53	Corman [41]	2020	2021	2022	#4
25	10	0.01	2.28	Patel [42]	2020	2021	2022	#3
26	9	0.00	2.28	Dagan [43]	2021	2021	2022	#5
27	26	0.04	0.00	Van [44]	2020	-	-	#0
28	25	0.02	0.00	Chu [45]	2020	-	-	#0
29	22	0.03	2.29	Trabucchi [46]	2020	2020	2020	#0
30	30	0.03	2.93	Wang [47]	2020	2020	2020	#1
31	29	0.06	0.00	McGitton [48]	2020	2020	2020	#1
32	24	0.02	1.92	ArMitage [49]	2020	2020	2020	#1
33	23	0.02	0.00	Williamson [50]	2020	-	-	#1
34	33	0.07	0.00	Brown [51]	2021	-	-	#2
35	32	0.01	0.00	Gorges [52]	2020	-	-	#2
36	26	0.00	2.26	CDC [53]	2020	2020	2020	#4
37	16	0.02	0.00	He [54]	2020	-	-	#4
38	32	0.09	0.00	Polack [55]	2020	-	-	#5
39	19	0.00	0.00	Baden [56]	2021	-	-	#5
40	19	0.03	0.00	Richardson [57]	2020	-	-	#6
41	31	0.02	0.00	Thompson [6]	2020	-	-	#7
42	22	0.00	0.00	Barnett [58]	2020	-	-	#7
43	19	0.00	0.00	Burton [59]	2020	-	-	#7
44	11	0.00	0.00	Zimmerman [12]	2020	-	-	#7
45	31	0.02	0.00	Grabowski [60]	2020	-	-	#8
46	11	0.00	2.18	Abbasi [61]	2020	2020	2020	#8
47	5	0.00	2.57	Chidambaram [62]	2020	2020	2020	#8
48	4	0.00	2.05	Grabowski [63]	2020	2020	2020	#8
49	3	0.00	1.54	Kaiser [64]	2020	2020	2020	#8

Note: # represents a knowledge cluster.

**Table 6:** Twenty-six representative keywords in terms of occurrences and centrality.

No.	Count	Centrality	Keyword	No.	Count	Centrality	Keyword
1	299	0.05	LTC	14	33	0.04	Social isolation
2	230	0.04	Nursing home	15	32	0.07	Facility
3	81	0.06	Older adult	16	32	0.02	Loneliness
4	72	0.01	COVID-19	17	30	0.05	Outbreak
5	68	0.02	LTC facility	18	29	0.03	Older people
6	59	0.05	Resident	19	28	0.05	Mortality
7	54	0.06	Impact	20	27	0.03	Dementia
8	52	0.06	Care	21	27	0.03	United States
9	48	0.04	Health	22	26	0.07	Mental health
10	40	0.05	Infection	23	26	0.02	Home
11	39	0.08	People	24	25	0.02	Prevalence
12	35	0.03	Risk	25	25	0.02	Public health
13	35	0.03	Quality	26	16	0.10	Nursing home residents

## Data Availability

The data that support the findings of this study is publicly available and comes from Web of Science.
